# Identification of a novel candidate locus at 21q22.3 for isolated intestinal Malrotation

**DOI:** 10.1093/hmg/ddag114

**Published:** 2026-10-31

**Authors:** Nadav Agam, Nitza Newman, Vadim Dolgin, Ofek Freund, Matan M Jean, Amit Safran, Tomer Poleg, Zaki Assi, Ohad S Birk

**Affiliations:** Morris Kahn Laboratory of Human Genetics, National Institute of Biotechnology in the Negev, Ben Gurion University, Ben-Gurion Blvd. 1, Be'er Sheva 8410501, Israel; Faculty of Health Sciences, Ben Gurion University of the Negev, Ben-Gurion Blvd. 1, Beer Sheva 8410501, Israel; Faculty of Health Sciences, Ben Gurion University of the Negev, Ben-Gurion Blvd. 1, Beer Sheva 8410501, Israel; Department of Pediatric Surgery, Soroka University Medical Center, Yitzhak Rager Blvd. 151, Beer Sheva 8457108, Israel; Morris Kahn Laboratory of Human Genetics, National Institute of Biotechnology in the Negev, Ben Gurion University, Ben-Gurion Blvd. 1, Be'er Sheva 8410501, Israel; Faculty of Health Sciences, Ben Gurion University of the Negev, Ben-Gurion Blvd. 1, Beer Sheva 8410501, Israel; Genetics Institute, Soroka University Medical Center, Yitzhak Rager Blvd. 151, Be'er Sheva 8457108, Israel; Morris Kahn Laboratory of Human Genetics, National Institute of Biotechnology in the Negev, Ben Gurion University, Ben-Gurion Blvd. 1, Be'er Sheva 8410501, Israel; Faculty of Health Sciences, Ben Gurion University of the Negev, Ben-Gurion Blvd. 1, Beer Sheva 8410501, Israel; Morris Kahn Laboratory of Human Genetics, National Institute of Biotechnology in the Negev, Ben Gurion University, Ben-Gurion Blvd. 1, Be'er Sheva 8410501, Israel; Faculty of Health Sciences, Ben Gurion University of the Negev, Ben-Gurion Blvd. 1, Beer Sheva 8410501, Israel; Morris Kahn Laboratory of Human Genetics, National Institute of Biotechnology in the Negev, Ben Gurion University, Ben-Gurion Blvd. 1, Be'er Sheva 8410501, Israel; Faculty of Health Sciences, Ben Gurion University of the Negev, Ben-Gurion Blvd. 1, Beer Sheva 8410501, Israel; Morris Kahn Laboratory of Human Genetics, National Institute of Biotechnology in the Negev, Ben Gurion University, Ben-Gurion Blvd. 1, Be'er Sheva 8410501, Israel; Faculty of Health Sciences, Ben Gurion University of the Negev, Ben-Gurion Blvd. 1, Beer Sheva 8410501, Israel; Faculty of Health Sciences, Ben Gurion University of the Negev, Ben-Gurion Blvd. 1, Beer Sheva 8410501, Israel; Department of Pediatric Surgery, Soroka University Medical Center, Yitzhak Rager Blvd. 151, Beer Sheva 8457108, Israel; Morris Kahn Laboratory of Human Genetics, National Institute of Biotechnology in the Negev, Ben Gurion University, Ben-Gurion Blvd. 1, Be'er Sheva 8410501, Israel; Faculty of Health Sciences, Ben Gurion University of the Negev, Ben-Gurion Blvd. 1, Beer Sheva 8410501, Israel; Genetics Institute, Soroka University Medical Center, Yitzhak Rager Blvd. 151, Be'er Sheva 8457108, Israel; The Danek Gertner Institute of Human Genetics, Sheba Medical Center, Derech Sheba 2, Ramat Gan 5262000, Israel

**Keywords:** intestinal Malrotation, autosomal recessive, 21q22.3, primary cilia, left–right patterning, *PCNT*

## Abstract

Intestinal malrotation (IM) is a relatively common congenital anomaly of the gastrointestinal tract resulting from abnormal rotation during embryonic development. While some cases of IM are part of syndromic manifestations, most are isolated, with their genetic basis remaining largely unknown. We present a familial case of apparently autosomal recessive isolated IM in a consanguineous kindred of Arab-Bedouin descent. Nine individuals from two nuclear families were recruited. Homozygosity mapping and logarithm of odds (LOD) score calculations were performed using single nucleotide polymorphism (SNP) array data. Whole-exome and whole-genome sequencing data were analyzed to identify candidate variants. Various analysis and interpretation tools were applied to evaluate and describe potential disease-associated variants. Homozygosity mapping identified a 2 Mb candidate locus on chromosome 21q22.3. Multipoint linkage analysis identified an overlapping locus on chromosome 21 (maximal LOD score: 3.168). A missense variant in PCNT (Gln1951Pro), along with several other segregating candidates, was evaluated. Our findings demonstrate a novel candidate locus at chromosome 21q22.3 associated with isolated IM, with *PCNT* emerging as a potential candidate gene. Further studies involving additional affected families and functional validation are required to establish the contribution of this locus and the identified variant to the pathogenesis of isolated IM.

## Author Summary

**Question:** What genetic factors underlie isolated intestinal malrotation (IM)?

**Finding:** In a consanguineous family with multiple affected members, homozygosity mapping and linkage analysis identified a novel candidate locus on chromosome 21q22.3 (maximum LOD score: 3.168). A segregating missense variant in *PCNT* was identified, highlighting it as a potential candidate gene.

**Meaning:** These findings provide additional evidence for a genetic contribution to isolated IM and identify a novel candidate locus and gene for further investigation. Validation in additional affected families and functional studies will be required to establish their role in disease pathogenesis.

## Introduction

Intestinal malrotation (IM, OMIM identifier: 193250, HPO: HP:0002566) is a potentially life-threatening, yet elusive, congenital anomaly [1]. As intestinal rotational anomalies vary, their severity depends on the likelihood of complications such as midgut volvulus and intestinal obstruction, with some being less dangerous than others [2]. Mild forms may be asymptomatic and are often identified as incidental radiological findings, typically requiring semi-elective laparotomy and Ladd’s operation repair. In contrast, symptomatic IM occurs in approximately 1 in 500 live births, with 90% of cases diagnosed within the first year of life, usually presenting with bilious vomiting and peritoneal signs [3, 4].

The pathogenesis of this condition arises from an abnormal sequence of maneuvers by the intestinal loop around the axis of the superior mesenteric artery, occurring between the fourth and tenth weeks of gestation [5]. During this process, the herniated intestinal loop fails to complete its 270° counterclockwise rotation. Interestingly, nonrotation (namely, a complete lack of rotation) tends to result in fewer complications compared to the intermediate state of malrotation. The coexistence of a non-rotating duodenojejunal loop limb and a partially rotating cecocolic loop limb in the mid-upper abdomen leads to a narrow mesenteric base, increasing the likelihood of volvulus and acute ischemia of the superior mesenteric artery and vein [6]. Additionally, the pressure exerted on the duodenum by the peritoneal Ladd’s bands— which fix the mispositioned cecum to the abdominal wall—can cause extrinsic compression and even obstruction. This scenario is potentially fatal and may lead to an inability to absorb proteins and fats, forcing these typically young patients to permanently depend on parenteral nutrition [7].

While the anatomical, physiological, and clinical features of IM are well characterized, the molecular mechanisms underlying these traits remain largely unclear. Rotational anomalies may present with additional congenital conditions, including diaphragmatic hernia, cardiac defects, and omphalocele, although associations with various gastrointestinal anomalies are also observed [2]. Such syndromic presentations may have an established molecular basis, potentially shedding light on the disease’s etiology. However, they may also obscure it, as the altered developmental landscape could create a significantly different context. Furthermore, various modes of inheritance have been reported in familial cases of IM, highlighting its prominent yet diverse genetic component [5]. Although most rotational anomalies are isolated, no disease-associated loci or genes have been identified to date.

The diagnosis of developmental syndromes often leads to comprehensive examination, increasing the likelihood of identifying IM and preventing complications. However, isolated IM is cryptic and may not be recognized until it becomes clinically acute and dangers. We present a familial case of isolated IM in a consanguineous kindred of Arab Bedouin descent. Following genetic analysis, we report a disease-associated locus and highlight potential genetic etiologies of isolated IM. Although the genetic basis of isolated intestinal malrotation remains incompletely understood, a recent genome-wide association study by Gaitanidis et al. identified a locus at chromosome 17p13 associated with intestinal malrotation [8]. These molecular findings add to the cumulating evidence regarding isolated IM, enhance our understanding of its underlying biology and offer potential strategies for preventing future cases.

## Results

### Clinical features

Affected individual IV-3 was born at 35 + 2 weeks of gestation following inadequate prenatal care (Fig. 1A). At the age of two days, she underwent a bowel obstruction workup with a differential diagnosis of Hirschsprung’s disease or meconium ileus. However, a contrast enema study was normal, and a rectal biopsy, which was later found to be normal, was performed. At 23 days of age, small bowel obstruction was diagnosed after the onset of static vomiting. IM, with a wide bowel loop located 35 cm from the ileocecal valve, was identified during an exploratory Ladd’s operation.

**Figure 1:**
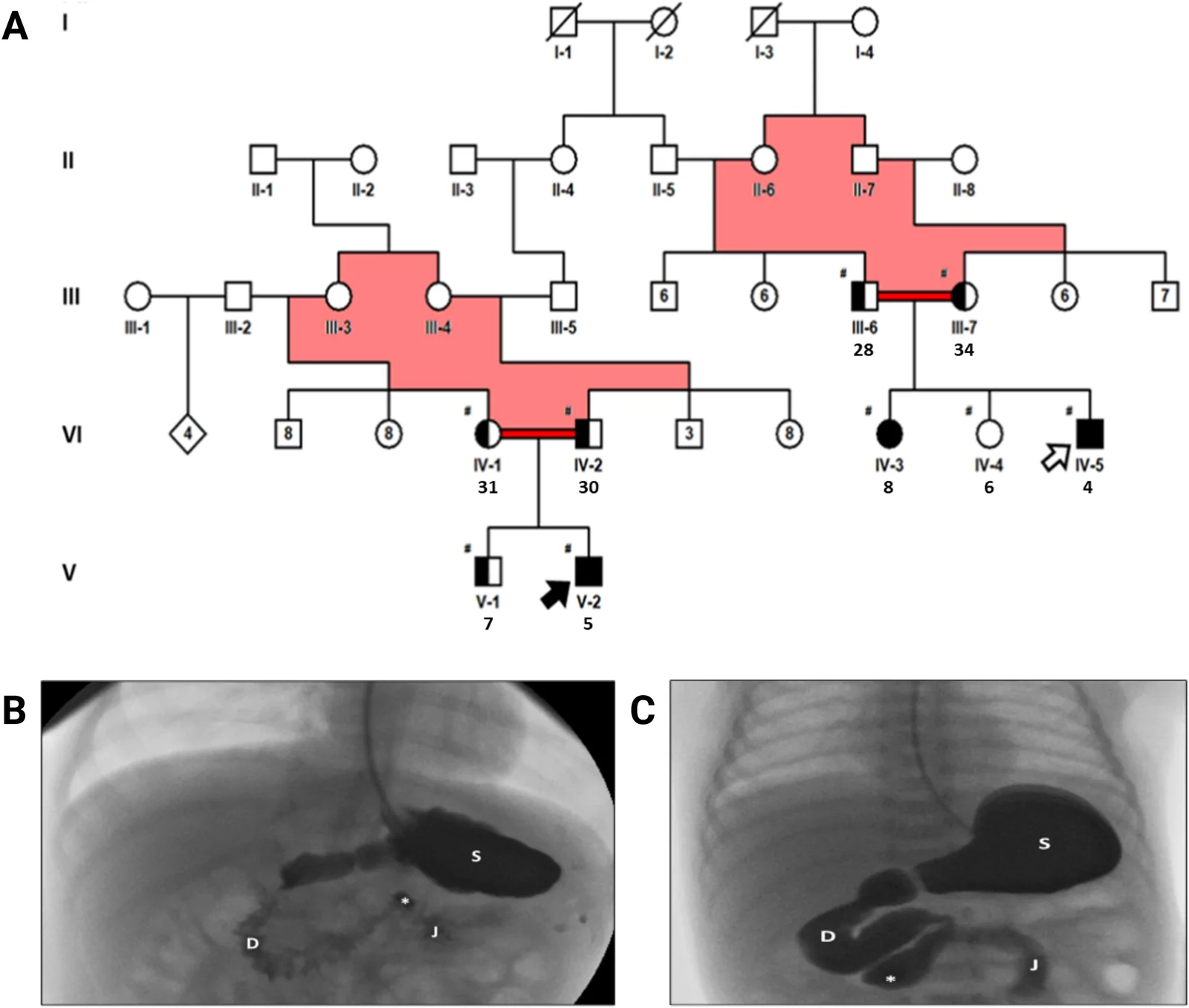
Characterization of the queried kindred. a; a pedigree, encompassing both branches, roman numerals represent generation, # denotes individuals that have been sampled and included in the analysis, arrow heads denote NGS, hollow arrow—WES, filled arrow—WGS, highlighted polygons emphasize consanguineous relations. B; an abdominal X-ray of healthy, unrelated individual. C; a representative abdominal X-ray of individual V-2, demonstrating the characteristic ‘corkscrew’ sign.

Affected individual IV-5 is the younger brother of IV-3 and was born at 39 weeks following an uneventful pregnancy and infancy of his older, unaffected sister, IV-4 (Fig. 1A). After experiencing vomiting after every feed, he was admitted to the pediatric emergency unit at 11 days of age. A contrast study of the upper gastrointestinal tract, followed by an emergency laparotomy, revealed a 180-degree volvulus and IM, without evidence of bowel ischemia.

Affected individual V-2 belongs to a different branch within the queried Bedouin tribe and was born at 36 weeks of a pregnancy with no prenatal care (Fig. 1A and C). He was admitted at 6 days of age with symptoms of shock, bloody stools, abdominal distention, and tenderness. A contrast upper gastrointestinal study, followed by an emergency laparotomy, revealed an IM and a 720-degree volvulus, including a section of ischemic bowel.

In all cases, once the diagnosis was established, Ladd’s procedure was performed. After an uneventful recovery in the hospital, all affected individuals were discharged on a full oral feeding regimen.

### Linkage analysis

Homozygosity mapping identified two homozygous loci (Fig. 2A-D): a ~ 2 Mbp locus on the long arm of chromosome 21 (21q22.3, rs2838588-rs117164191, 21:44557228–46 665 705, hg38 genome build, contains the following protein coding genes: *KRTAP10–3, KRTAP10–4, KRTAP10–5, KRTAP10–6, KRTAP10–7, KRTAP10–8, KRTAP10–9, KRTAP10–10, KRTAP10–11, KRTAP10–12, KRTAP12–1, KRTAP12–2, KRTAP12–3, KRTAP12–4, TSPEAR, UBE2G2, SUMO3, PTTG1IP, ITGB2, SLX9, ADARB1, POFUT2, COL18A1, SLC19A1, PCBP3, COL6A1, COL6A2, FTCD, SPATC1L, LSS, MCM3AP, YBEY, C21orf58, PCNT, DIP2A, S100B, and PRMT2*) and a shorter, ~ 650 Kbp locus on chromosome 4 (4q34.2-4q34.3, rs4690696-rs13112548, 4:176393646–177 052 908, contains one protein coding gene: *VEGFC*). Genome-wide multipoint LOD score calculation revealed a ~ 1.4 Mb locus on chromosome 21 that overlapped with the one identified by homozygosity mapping (rs915876-rs2245760, 21:43956326–45 376 207), with a maximal score of 3.168 (see Fig. 2E-F, output of all other chromosomes will be provided upon request.

**Figure 2:**
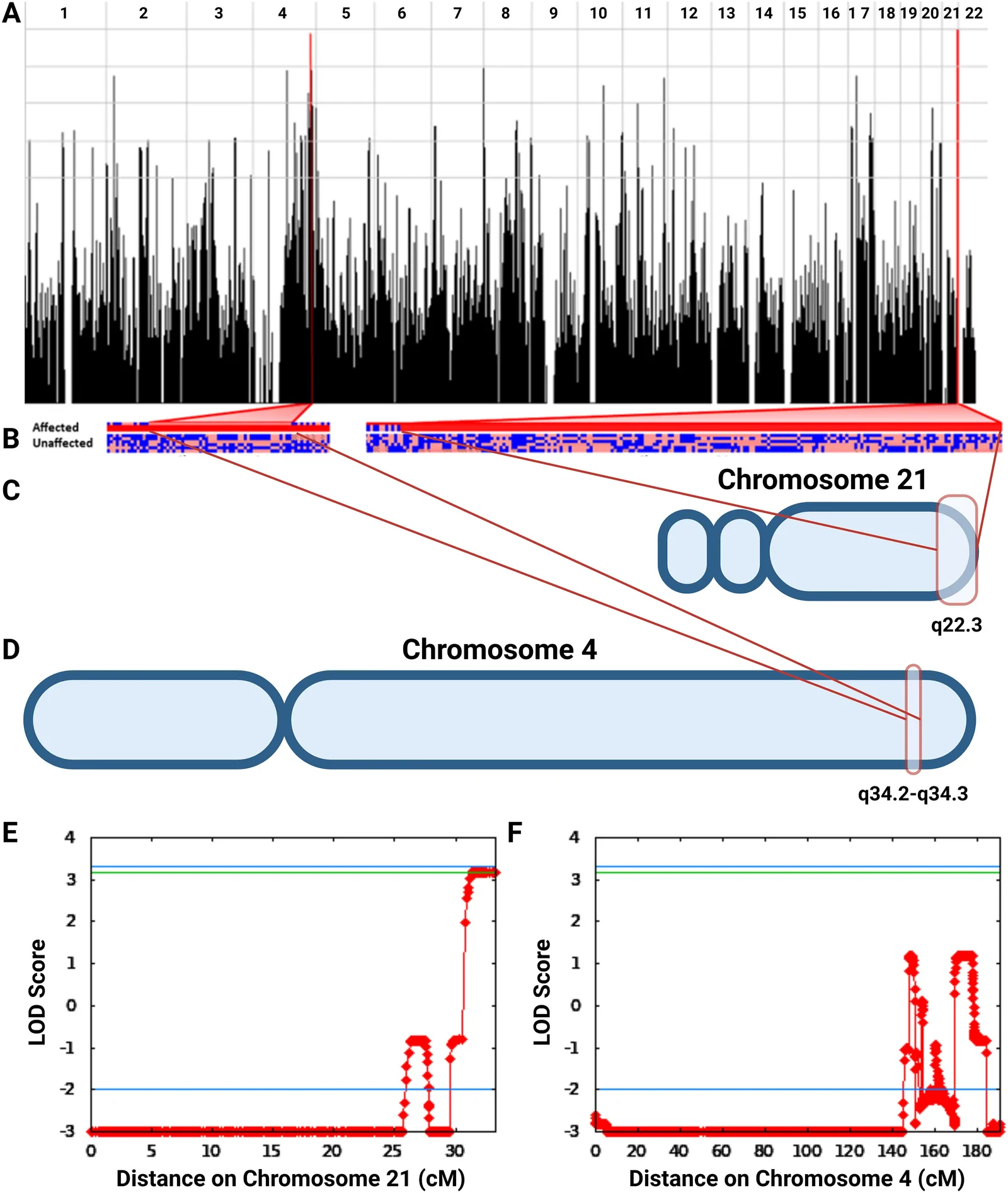
Genome-wide linkage analysis of SNP array data. A; a Manhattan plot, showing homozygosity mapping. Numbers above denote the examined chromosome in each column, black bars denote the relative compatibility of each locus with the expected genotype of each individual. Red bars denote homozygous loci in all affected individuals, but not in any unaffected. B; rectangles denote individual genotypes for each SNP (affected (n = 3) above, and unaffected (n = 6) below): Red and blue denote homozygous and heterozygous, respectively. C, D; a schematic representation of chromosomes 21 and 4, respectively. Red rectangles denote disease-associated loci. E, F; multi-point LOD score calculation of chromosomes 21 and 4, respectively, delineating an overlapping, disease-associated locus in chromosome 21.

### Variants filtration

Analysis of NGS data in the Franklin platform identified a total of 28 variants, 13 of which were classified as batch effects or found in a homozygous state in healthy controls from our in-house database (see Supplementary materials—appendix 3). Of the remaining variants, 13 had a CADD score of 10 or below (the suggested cutoff for identifying pathogenic variants, https://cadd.gs.washington.edu/info), and were therefore excluded, leaving two variants for further investigation (data given per GRCh38).

The first variant was a missense mutation, Gln1951Pro, in the *PCNT* gene (HGVS: NC_000021.9:g.46411925A > C), with a CADD score of 22.4 and a PhyloP score of 2.057 (conserved in mammals). The second variant was a missense mutation, Ser172Pro, in the *KRTAP10–11* gene (HGVS: NC_000021.9:g.44646972 T > C), with a CADD score of 16.59 and a PhyloP score of 0.051. In addition to its missense effect, the *KRTAP10–11* variant is also an intronic variant in the *TSPEAR* gene, as the entire *KRTAP10–11* gene resides within one of its introns. Since the *KRTAP10–11* variant is not conserved in many mammals and the gene is expressed almost exclusively in the skin (GTEx; https://gtexportal.org/home/gene/KRTAP10-11, Evo-devo; https://apps.kaessmannlab.org/evodevoapp/). [9, 10]), it received low priority. Ultimately, we identified one potentially pathogenic variant in the *PCNT* gene.

Analysis of WGS data for structural variants identified a total of 37 variants, 29 of which were caused by batch effects or were heterozygous (see Supplementary materials—appendix 4). Of the remaining eight, seven were excluded due to the identification of healthy homozygotes in our in-house database. The last variant was queried in the GnomAD SVs database, where healthy homozygotes were also found, and was subsequently excluded as well.

### Variant assessment

The region surrounding the *PCNT* variant was sequenced in all available samples, and its segregation across the pedigree was validated (Fig. 3A, supplementary materials—appendix 5), as expected in a recessive mode of inheritance. The glutamine residue at position 1951 is conserved across mammals and has remained unchanged from mice to humans (Fig. 3B).

**Figure 3:**
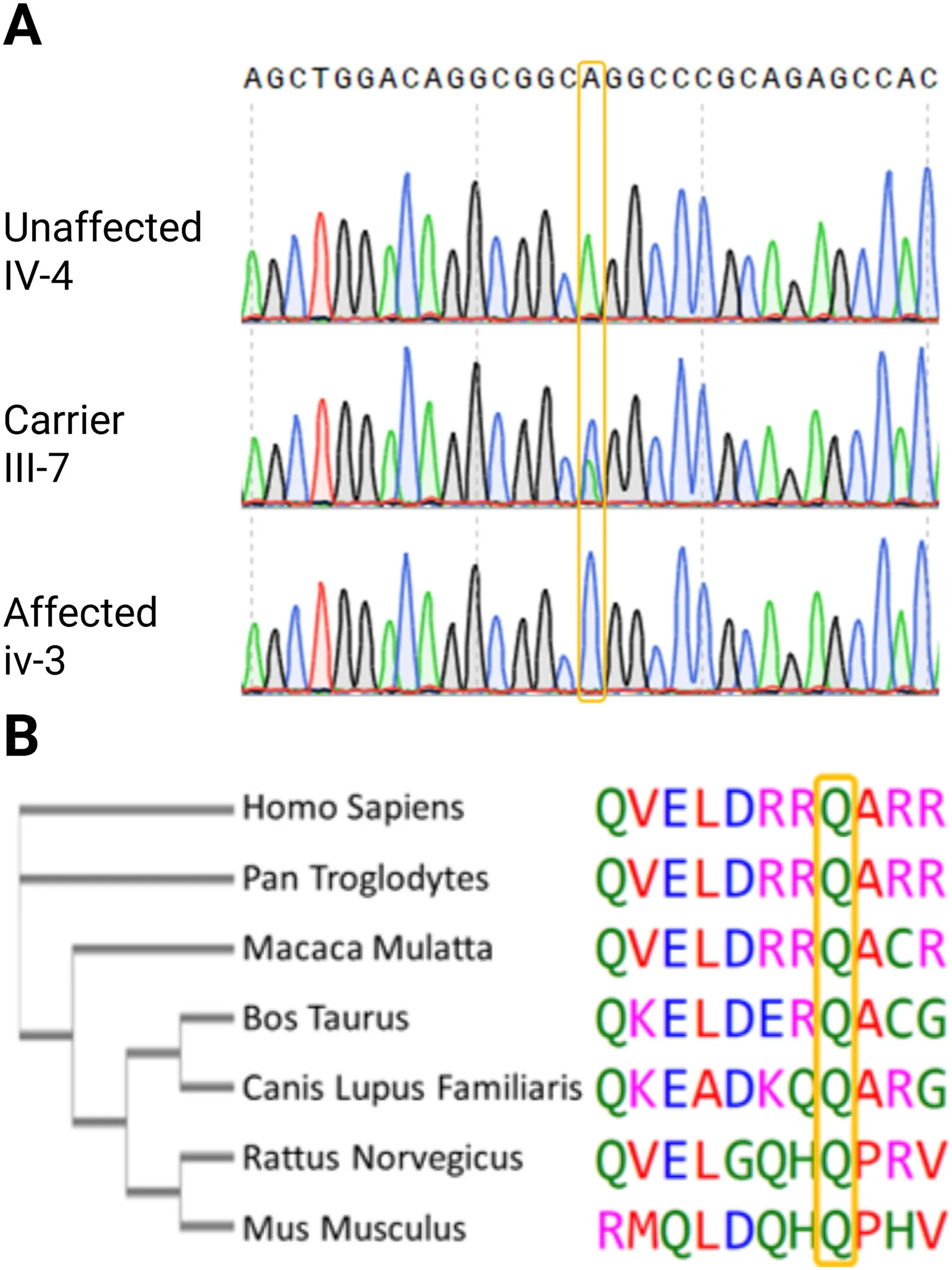
Evaluation of the *PCNT* variant. A; Sanger sequencing of unaffected, unaffected-carrier, and affected individuals. Rectangle denotes the mutated position. B; Evolutionary conservation across mammalian species of the mutated glutamine at position 1951 and surrounding region. Rectangle denotes the mutated position.

## Discussion

We report on a familial case of autosomal recessive isolated IM. Genome-wide linkage analysis identified two disease-associated loci, with one showing a significant degree of likelihood. Analysis of NGS data revealed a candidate variant in the *PCNT* gene, which exhibits pathogenic potential, based on frequency, evolutionary conservation, and prediction tools. Of note, IM encompasses a heterogeneous spectrum of anatomical abnormalities that may arise through different developmental and genetic mechanisms. Accordingly, our findings should be interpreted in the context of the specific familial phenotype investigated here, rather than as evidence for a common genetic basis underlying all cases of isolated intestinal malrotation. Although the three affected individuals exhibited variable clinical presentations and degrees of intestinal volvulus, the occurrence of multiple cases of this relatively uncommon congenital anomaly within the same extended consanguineous kindred warranted investigation using a family-based genetic approach. Furthermore, the affected individuals did not exhibit additional clinical manifestations suggestive of a recognizable syndromic disorder. In particular, no associated cardiovascular, pulmonary, neurological, or skeletal abnormalities were reported, which are characteristic of several established syndromic conditions associated with intestinal malrotation. Nevertheless, the possibility of an underlying genetic syndrome cannot be entirely excluded, and our findings should be interpreted in the context of the specific familial phenotype investigated.

Although all affected individuals, and none of the unaffected ones, were homozygous for both queried loci, the maximal logarithm of odds (LOD) score was calculated only for the locus on chromosome 21 (see Fig. 2E and F). Thus, the chromosome 4 locus should be regarded as a preliminary finding, as its association with isolated IM remains unconfirmed.

Given the high degree of consanguinity within the studied Arab-Bedouin kindred, homozygosity mapping is particularly suitable for identifying recessive disease-associated loci. However, extensive consanguinity also increases background homozygosity, potentially reducing the specificity of this approach by increasing the likelihood of identifying shared homozygous regions unrelated to the phenotype. Additionally, technical factors, such as variations in locus or chromosome length and the number of informative markers, may influence the resolution of linkage analysis. To mitigate the limitations imposed by the relatively small number of affected individuals, we included as many available affected and unaffected family members as possible in the genetic analyses and cross-referenced the results obtained using two independent linkage/homozygosity mapping platforms. Furthermore, candidate variants were evaluated against our in-house database, which includes several hundred samples, including ethnically matched individuals, to reduce the likelihood of prioritizing variants that are homozygous in unaffected individuals from the same population. Nevertheless, these complementary approaches cannot fully overcome the limited statistical power associated with the small pedigree, and further validation in additional affected families is warranted. Nevertheless, validation in an independent cohort of affected individuals would have further strengthened our findings; however, such validation was not feasible, as we are not aware of any additional familial cases of isolated intestinal malrotation suitable for replication of our results. Finally, although structural variant analysis was performed using short-read whole-genome sequencing data, the sensitivity of this approach is not absolute, and certain types of structural variants, may have remained undetected.

Several insights into the molecular basis of various syndromes with multi-organ phenotypes, including IM, have been reported. A syndromic form of IM, characterized by alveolar capillary dysplasia, misalignment of the pulmonary veins, and other associated features, is caused by truncating mutations in the *FOXF1* gene [11]. As *FOXF1* regulates mesodermal derivatives, it is believed to play a crucial role in the formation of the dorsal mesentery, a key factor in ensuring proper intestinal rotation [12, 13]. Another syndromic form, which includes pyloric stenosis, undescended testicles, and central nervous system involvement, is associated with aberrant function of *FLNA*. *FLNA* encodes an actin-binding protein that regulates cell shape and migration [14, 15].

IM has also been reported in association with several left–right patterning-related syndromes, all of which involve both the gastrointestinal and cardiovascular systems, as well as other organs. The known genes associated with these pathologies include *CFC1*, *ZIC3*, *NKX2–5, ACVR2B*, and *LEFTY2*, all of which play roles in developmental processes such as mesodermal commitment, TGF-β signaling, and left–right patterning [16–26]. Notably, all the discussed genes cause disease through an autosomal-dominant mode of inheritance, except for mutations in *FLNA* and *ZIC3*, which are inherited in an X-linked recessive pattern [5]. Interestingly, ITGB2, located within the chromosome 21q22.3 candidate region, encodes integrin β2, which has been reported to interact with filamin A (FLNA), an actin-binding protein implicated in intestinal developmental and motility disorders [27].

Finally, analysis of sporadic cases of syndromic IM has recently linked it to several rare copy number variants, some of which are located within loci previously associated with the disease [28].

Non-syndromic familial cases of IM are rare, with only two reported instances [5, 29, 30]. In line with the syndromic manifestation, both reports describe an autosomal-dominant mode of inheritance, although no genetic analysis was performed. However, clinical descriptions of syndromic IM inherited in an autosomal-recessive pattern have been published, along with several chromosomal aberrations observed in sporadic cases [5, 31–34]. Thus, the identification of the linked loci and the discussed candidate constitute the first genetic insight into isolated IM.

PCNT, or Pericentrin, an integral component of the pericentriolar material and the filamentous matrix of the centrosome, plays a key role in the initial assembly of microtubule organization during cell division [35, 36]. A calmodulin-binding protein, pericentrin interacts with PCM1 and DISC1 and is involved in the formation of the microtubule network. [37–39]. As such, disruption of *PCNT* activity results in disorganized mitotic spindles, missegregation of chromosomes, and prevention of serum starvation-induced primary cilium formation [39–41]. Notably, PCNT has been found to be essential for primary cilia formation through its binding to PKD2 and intraflagellar transport proteins, such as IFT20 [39, 42].

The canonical presentation of PCNT complete loss-of-function in humans is microcephalic osteodysplastic primordial dwarfism type II (MOPD2), which follows an autosomal recessive mode of inheritance [41]. A multi-organ disease, this developmental syndrome is characterized by intrauterine growth retardation, severe short stature, and microcephaly [43]. Additional symptoms include café-au-lait spots, Moyamoya disease or other cerebral neurovascular abnormalities, and coronary artery hypoplasia [44, 45]. Importantly, the vast majority of reported disease-causing variants result in a complete loss of function of PCNT (e.g. introduction of a stop codon, frameshift-causing indels, etc. [43], and Qiagen’s HGMD database). One possible explanation for this phenotypic difference is that the identified missense variant may retain partial PCNT function, potentially resulting in a milder phenotype. However, no functional studies were performed to assess the effect of Gln1951Pro on PCNT activity, and its potential hypomorphic nature remains speculative.

Other syndromes associated with PCNT include Seckel syndrome and Majewski type III, which present with partially overlapping symptoms [46]. Interestingly, a case report of Majewski syndrome with IM has been published [47]. Notably, the phenotype observed in our affected individuals differs substantially from the classical multisystem manifestations of PCNT-associated syndromes. This phenotypic discrepancy warrants caution when interpreting the potential involvement of the identified PCNT variant in isolated intestinal malrotation. Interestingly, *YWHAE*, one of the genes highlighted by Gaitanidis et al. in their chromosome 17p13 association study, is also involved in cell-cycle and cytoskeletal regulation, suggesting a potential functional overlap with *PCNT*, although a direct molecular interaction between the two has not been established [8, 48]. Although the established roles of PCNT in centrosomal and ciliary function, together with previous reports linking related developmental disorders to intestinal malrotation, provide biological plausibility for its involvement, this evidence remains indirect and does not establish a causal role for PCNT in isolated IM.

Defects in left–right patterning are caused by various molecular mechanisms; a prominent one is impaired primary cilia function [49]. In agreement, the genetic causes of primary ciliary dyskinesia and those of situs ambiguous overlaps [49]. Notably, a recent report has described several mutations linking *PCNT* to heterotaxy [50]. Notably, out of a total of thirty-eight protein coding genes within the two disease-associated loci we identified, only *PCNT* is involved in primary cilia formation and function. Although the segregation pattern, evolutionary conservation, and predicted functional impact of the identified *PCNT* variant support its candidacy, these findings do not establish a causal relationship between *PCNT* and isolated IM in the absence of functional validation or replication in additional affected families.

Isolated IM is a cryptic yet relatively common congenital defect with serious, potentially fatal complications. Therefore, developing means to diagnose it and enable preventive measures is essential. Deciphering its underlying mechanisms, or at least its genetic patterns, may help achieve this goal. Conclusively, further studies are needed to characterize disease-associated loci in this context, focusing on identifying disease-causing genes and specific IM-associated SNPs, as well as substantiating the involvement of *PCNT* in its pathogenesis.

## Materials and methods

### Clinical assessment, recruitment, and DNA purification

All clinical diagnoses were performed by pediatric surgeons, and geneticists in clinical settings. A total of nine individuals from two nuclear families of Arab-Bedouin ethnicity with affected members (see Fig. 1) were recruited and formal written consent was obtained (following approval from the Institutional Review Board of Soroka Medical Center—approval No. 5071G). DNA was extracted from peripheral blood using the E.Z.N.A® SQ Blood DNA Kit (OMEGA Bio-Tek, SKU: D5032–03), according to the manufacturer’s instructions.

### Single nucleotide polymorphism (SNP) Array and linkage analysis

Single nucleotide polymorphism (SNP) array analysis was conducted using the Illumina Omni Express BeadChip platform, which includes over 750 000 SNP loci (Illumina Inc., San Diego, CA, USA). Homozygosity mapping (Fig. 2A-D) was performed using the HomozygosityMapper web tool (https://www.homozygositymapper.org/). Genome-wide LOD scores were calculated using the Superlink online software (http://cbl-hap.cs.technion.ac.il/superlink-snp/) (Figure 2,E–F). Analysis parameters are provided in supplementary materials—appendices 1 and 2, SNP array data will be provided upon request.

### Next generation sequencing (NGS) and variants filtration

Whole-exome sequencing (WES, HiSeq 2500, Illumina, San Diego, CA) and whole-genome sequencing (WGS, NovaSeq 6000, Illumina, San Diego, CA) were performed on one affected individual from each branch, using a paired-end (2 × 100) protocol by Macrogen (Korea). Read alignment, variant calling, and annotation were conducted by the manufacturer using BWA, GATK, and SnpEff, respectively (NGS data will be provided upon request).

NGS data was analyzed using Genoox’s Franklin® software (https://franklin.genoox.com), with standard parameters for autosomal recessive inheritance. It was further examined with the Combined Annotation Dependent Depletion (CADD) tool (https://cadd.gs.washington.edu/), PhyloP scores for phylogenetic p-values, and our in-house VARista platform [51], which incorporates data from approximately 800 NGS samples, ~ 50% of which ethnically match the queried family (variant filtration pipeline and full list of variants available in supplementary materials—appendix 3). NGS data from both patients was analyzed to identify shared variants, in addition to separate WGS analysis to retain non-coding variants absent in the WES data. All variants were verified using the Integrative Genomics Viewer (IGV) software (Broad Institute, https://software.broadinstitute.org/software/igv/).

Additionally, structural variants were identified using the Manta software, a structural variant caller developed by Illumina (San Diego, CA, [52]). The output was subsequently compared against our in-house database and the GnomAD SVs database (https://gnomad.broadinstitute.org/). (structural variant filtration pipeline and full list of structural variants available in supplementary materials—appendix 4).

To identify potential sequencing batch-associated artifacts, candidate single-nucleotide variants and structural variants (SVs) were cross-referenced against all available samples from unrelated, unaffected individuals sequenced in the same WGS batch. Variants consistently detected in all examined samples within the batch were considered putative batch-associated artifacts and excluded from further candidate prioritization.

### Variant assessment

The co-segregation of the queried PCNT variant across all available individuals within the studied pedigree was validated using Sanger sequencing (primers sequences and PCR parameters available in in Supplementary materials—appendix 5). Evolutionary conservation was demonstrated using the Clustal Omega multiple sequence alignment tool (https://www.ebi.ac.uk/Tools/msa/clustalo/) and protein FASTA sequences obtained from NCBI (see supplementary materials—appendix 6).

## Patient consent

The authors confirm that patient consent forms have been obtained for this study.

## Supplementary Material

Supplementary_Materials_Agam_et_al_HMG-26-00517_ddag114

## Data Availability

All the required data used in this study, including formatted inputs and and tools, are available in the Supplemental Materials. Additional data, if required, will be provided upon request.
