# Supplementary material for: Identification of a novel candidate locus at 21q22.3 for isolated intestinal Malrotation
Source: Hum Mol Genet. 2026 Oct 31;35(22):ddag114. doi: 10.1093/hmg/ddag114 (PMC13723271; doi:10.1093/hmg/ddag114)
Supplement: Supplementary file 1 [file supplementary_materials_agam_et_al_hmg-26-00517_ddag114.docx]

**Supplementary Material**

**Table of contents**

Appendix 1 – Homozygosity mapping parameters and output.

Appendix 2 – Linkage analysis parameters and output.

Appendix 3 – Franklin software output, variant list of variants within the queried loci.

Appendix 4 – Manta software output, structural variant list of variants within queried loci.

Appendix 5 – PCR reaction parameters for the *PCNT* variant.

Appendix 5 – Clustal Omega/multiple sequence alignment input.

**Appendix 1**

**Homozygosity mapping analysis of SNP array data using the HomozygosityMapper software**

**UPLOAD GENOTYPES PAGE:**

CHIP: "ILLUMINA: ILLUMINA (ANY ARRAY NOT LISTED HERE)"

GENOTYPE FILE: "HomozygosityMapper_input.txt"

CHECK BOX: "Illumina file with real genotypes, coded as A/C/G/T - due to the conversion into A/B genotypes, this is relatively slow." – CHECK

Minimum coverage: 10

USE…: "KNOWN VARIANTS PLUS VARIATIONS FROM REFSEQ"

**(RE)ANALYSE YOUR GENOTYPES**

CASES:

IV-3, IV-5, V-2

CONTROLS:

III-6, III-7, IV-1, IV-2, IV-4, V-1

CHECK BOX: "require genetic homogeneity" - CHECK

Exclude homozygous stretches in controls >= **300**

Allele frequencies: "none"

Limit block length = **50**

Only count blocks longer than: LEAVE EMPTY

**PROJECT PAGE**

THRESHOLD = O.9 x MAX

**Homozygosity mapping output (homozygous disease-associated loci):**

4:176393646-177052908 (HG38)

rs4690696 - rs13112548

21:44557228-46665705 (HG38)

rs2838588 - rs117164191

**Appendix 2**

**Multipoint LOD score calculation using the SuperLink software:**

**THE WIZARD > "CHOOSE METHOD SECTION"**

Choose an input format: "PED and SNP files"

**THE WIZARD > "UPLOAD FILES"**

Upload your snp file(s): "SuperLink_input.snp"

CHECK BOX: "Upload your ped file" –UNCHECK

CHECK BOX: "Pedigree in simplified input format:"

TEXT BOX (INPUT FORMAT):

pedigree: 1

I-3 0 0 1 1

I-4 0 0 2 1

II-5 0 0 1 1

II-6 I-3 I-4 2 1

II-7 I-3 I-4 1 1

II-8 0 0 2 1

III-6 II-5 II-6 1 1

III-7 II-7 II-8 2 1

IV-3 III-6 III-7 2 2

IV-4 III-6 III-7 2 1

IV-5 III-6 III-7 1 2

pedigree: 2

II-1 0 0 1 1

II-2 0 0 2 1

III-2 0 0 1 1

III-3 II-1 II-2 2 1

III-4 II-1 II-2 2 1

III-5 0 0 1 1

IV-1 III-2 III-3 2 1

IV-2 III-5 III-4 1 1

V-1 IV-2 IV-1 1 1

V-2 IV-2 IV-1 1 2

**THE WIZARD > "MAPPING"**

Are all column labels correct? : "YES"

Please verify if correct or change if needed and confirm: "CONFIRM MAPPING"

**THE WIZARD > "SEND TO ANALYSIS"**

CHECK BOX: "Remove uninformative markers" – CHECK

Disease mutant gene frequency: "0.01" (DEFAULT)

MODE OF INHERITANCE: CHECK RECESSIVE

RECESSIVE – PENETRANCE CHOICES: 0.99 (DEFAULT)

**Multipoint LOD score calculation output (homozygous disease associated locus):**

21: 43956326-45376207 (HG38)

rs915876 - rs2245760

MAXIMAL LOD SCORE OF 3.168077 AT rs584681

**Appendix 3**

**NGS variant filtration using the Franklin software:**

FRANKLIN ANALYSIS OUTPUT: 28 VARIANTS ([see the online table of variants here](https://docs.google.com/spreadsheets/d/1IwdoBm5zcnyO59bWAt2LCGVSyaaouABp/edit?usp=sharing&ouid=102997287756051052275&rtpof=true&sd=true) (in Chrome))

1) EXCLUDE VARIANTS THAT ARE CAUSED BY BATCH EFFECT OR HOMOZYGOUS IN IN-HOUSE DATABASE: 13 VARIANTS EXCLUDED (REMAIN=15)
2) EXCLUDE VARIANTS WITH CADD SCORE < 10: 13 VARIANTS EXCLUDED (**REMAIN=2**)

**REMAINED: 2 VARIANTS**

**Appendix 4**

**WGS structural variant filtration using the Manta software:**

TOTAL NUMBER OF VARIANTS = 37 ([see the online table of variants here](https://docs.google.com/spreadsheets/d/1UazMh_qvSMgiXM16ChSWnjd5HJD4yboS/edit?usp=sharing&ouid=102997287756051052275&rtpof=true&sd=true) (in Chrome))

BATCH EFFECT EXCLUSION = 25 VARIANTS (12 VALID VARIANTS LEFT)

HETEROZYGOSITY EXCLUSION = 4 VARIANTS (8 HOMOZYGOTE VARIANTS LEFT)

EXCLUSION BASED ON UNAFFECTED HOMOZYGOTES IN IN-HOUSE DATABASE = 7 VARIANTS (1 VARIANTS LEFT)

EXCLUSION BASED ON HOMOZYGOTES IN GNOMAD SV = 1 (**0 VARIANTS LEFT**)

**­­Appendix 5**

**PCR amplification of the region around the *PCNT* mutation:**

FORWARD: 5'-tgaaagcaaaggaagcgacg-3' (USED ALSO FOR SANGER SEQUENCING)

REVERSE: 5'-ttcactgcgtactgacacgg-3'

Tm= 60⁰C

PRODUCT LENGTH= 594BP (CHR21:46411661-46412254, HG38)

**Appendix 6**

>HOMO SAPIENS

DSSHSEKKTAKRKGSAVDASVQEESPVTKEDSALCGGGDIC

KSTSCDDTPDGAGGAFAAQPEDCDGEKREDLEQLQQKQVNDHPPEQCGMFTVSDHPPEQHGMFTVGDHPP

EQRGMFTVSDHPPEQHGMFTVSDHPPEQRGMFTISDHQPEQRGMFTVSDHTPEQRGIFTISDHPAEQRGM

FTKECEQECELAITDLESGREDEAGLHQSQAVHGLELEALRLSLSNMHTAQLELTQANLQKEKETALTEL

REMLNSRRAQELALLQSRQQHELELLREQHAREKEEVVLRCGQEAAELKEKLQSEMEKNAQIVKTLKEDW

ESEKDLCLENLRKELSAKHQSEMEDLQNQFQKELAEQRAELEKIFQDKNQAERALRNLESHHQAAIEKLR

EDLQSEHGRCLEDLEFKFKESEKEKQLELENLQASYEDLKAQSQEEIRRLWSQLDSARTSRQELSELHEQ

LLARTSRVEDLEQLKQREKTQHESELEQLRIYFEKKLRDAEKTYQEDLTLLQQRLQGAREDALLDSVEVG

LSCVGLEEKPEKGRKDHVDELEPERHKESLPRFQAELEESHRHQLEALESPLCIQHEGHVSDRCCVETSA

LGHEWRLEPSEGHSQELPWVHLQGVQDGDLEADTERAARVLGLETEHKVQLSLLQTELKEEIELLKIENR

NLYGKLQHETRLKDDLEKVKHNLIEDHQKELNNAKQKTELMKQEFQRKETDWKVMKEELQREAEEKLTLM

LLELREKAESEKQTIINKFELREAEMRQLQDQQAAQILDLERSLTEQQGRLQQLEQDLTSDDALHCSQCG

REPPTAQDGELAALHVKEDCALQLMLARSRFLEERKEITEKFSAEQDAFLQEAQEQHARELQLLQERHQQ

QLLSVTAELEARHQAALGELTASLESKQGALLAARVAELQTKHAADLGALETRHLSSLDSLESCYLSEFQ

TIREEHRQALELLRADFEEQLWKKDSLHQTILTQELEKLKRKHEGELQSVRDHLRTEVSTELAGTVAHEL

QGVHQGEFGSEKKTALHEKEETLRLQSAQAQPFHQEEKESLSLQLQKKNHQVQQLKDQVLSLSHEIEECR

SELEVLQQRRERENREGANLLSMLKADVNLSHSERGALQDALRRLLGLFGETLRAAVTLRSRIGERVGLC

LDDAGAGLALSTAPALEETWSDVALPELDRTLSECAEMSSVAEISSHMRESFLMSPESVRECEQPIRRVF

QSLSLAVDGLMEMALDSSRQLEEARQIHSRFEKEFSFKNEETAQVVRKHQELLECLKEESAAKAELALEL

HKTQGTLEGFKVETADLKEVLAGKEDSEHRLVLELESLRRQLQQAAQEQAALREECTRLWSRGEATATDA

EAREAALRKEVEDLTKEQSETRKQAEKDRSALLSQMKILESELEEQLSQHRGCAKQAEAVTALEQQVASL

DKHLRNQRQFMDEQAAEREHEREEFQQEIQRLEGQLRQAAKPQPWGPRDSQQAPLDGEVELLQQKLREKL

DEFNELAIQKESADRQVLMQEEEIKRLEEMNINIRKKVAQLQEEVEKQKNIVKGLEQDKEVLKKQQMSSL

LLASTLQSTLDAGRCPEPPSGSPPEGPEIQLEVTQRALLRRESEVLDLKEQLEKMKGDLESKNEEILHLN

LKLDMQNSQTAVSLRELEEENTSLKVIYTRSSEIEELKATIENLQENQKRLQKEKAEEIEQLHEVIEKLQ

HELSLMGPVVHEVSDSQAGSLQSELLCSQAGGPRGQALQGELEAALEAKEALSRLLADQERRHSQALEAL

QQRLQGAEEAAELQLAELERNVALREAEVEDMASRIQEFEAALKAKEATIAERNLEIDALNQRKAAHSAE

LEAVLLALARIRRALEQQPLAAGAAPPELQWLRAQCARLSRQLQVLHQRFLRCQVELDRRQARRATAHTR

VPGAHPQPRMDGGAKAQVTGDVEASHDAALEPVVPDPQGDLQPVLVTLKDAPLCKQEGVMSVLTVCQRQL

QSELLLVKNEMRLSLEDGGKGKEKVLEDCQLPKVDLVAQVKQLQEKLNRLLYSMTFQNVDAADTKSLWPM

ASAHLLESSWSDDSCDGEEPDISPHIDTCDANTATGGVTDVIKNQAIDACDANTTPGGVTDVIKNWDSLI

PDEMPDSPIQEKSECQDMSLSSPTSVLGGSRHQSHTAEAGPRKSPVGMLDLSSWSSPEVLRKDWTLEPWP

SLPVTPHSGALSLCSADTSLGDRADTSLPQTQGPGLLCSPGVSAAALALQWAESPPADDHHVQRTAVEKD

VEDFITTSFDSQETLSSPPPGLEGKADRSEKSDGSGFGARLSPGSGGPEAQTAGPVTPASISGRFQPLPE

AMKEKEVRPKHVKALLQMVRDESHQILALSEGLAPPSGEPHPPRKEDEIQDISLHGGKTQEVPTACPDWR

GDLLQVVQEAFEKEQEMQGVELQPRLSGSDLGGHSSLLERLEKIIREQGDLQEKSLEHLRLPDRSSLLSE

IQALRAQLRMTHLQNQEKLQHLRTALTSAEARGSQQEHQLRRQVELLAYKVEQEKCIAGDLQKTLSEEQE

KANSVQKLLAAEQTVVRDLKSDLCESRQKSEQLSRSLCEVQQEVLQLRSMLSSKENELKAALQELESEQG

KGRALQSQLEEEQLRHLQRESQSAKALEELRASLETQRAQSSRLCVALKHEQTAKDNLQKELRIEHSRCE

ALLAQERSQLSELQKDLAAEKSRTLELSEALRHERLLTEQLSQRTQEACVHQDTQAHHALLQKLKEEKSR

VVDLQAMLEKVQQQALHSQQQLEAEAQKHCEALRREKEVSATLKSTVEALHTQKRELRCSLEREREKPAW

LQAELEQSHPRLKEQEGRKAARRSAEARQSPAAAEQWRKWQRDKEKLRELELQRQRDLHKIKQLQQTVRD

LESKDEVPGSRLHLGSARRAAGSDADHLREQQRELEAMRQRLLSAARLLTSFTSQAVDRTVNDWTSSNEK

AVMSLLHTLEELKSDLSRPTSSQKKMAAELQFQFVDVLLKDNVSLTKALSTVTQEKLELSRAVSKLEKLL

KHHLQKGCSPSRSERSAWKPDETAPQSSLRRPDPGRLPPAASEEAHTSNVKMEKLYLHYLRAESFRKALI

YQKKYLLLLIGGFQDSEQETLSMIAHLGVFPSKAERKITSRPFTRFRTAVRVVIAILRLRFLVKKWQEVD

RKGALAQGKAPRPGPRARQPQSPPRTRESPPTRDVPSGHTRDPARGRRLAAAASPHSGGRATPSPNSRLE

RSLTASQDPEHSLTEYIHHLEVIQQRLGGVLPDSTSKKSCHPMIKQ

>PAN TROGLOTYDES

DSSHSEKKTAKRKGSAVNASVQEESPVTKEDSALCGGGDIC

KSTSCDDTPDGAGGAFAAQPEDCDGEKREDLEQLQQKQVNDHPPEQCGMFTVSDHQPEQRGMFTISDHPG

EQRGMFTKECEQECELAITDLESGREDEAGLQQSQAVHGLELEALRLSLSNMHTAQLELTQANLQKEKET

ALTELREMLNSRRAQELALLQSRQQHELELLREQHAREKEEVVLRCGQEAAELKEKLQSEMEKNAQIVKT

LKEDWESEKDLCLENLRKELSAKHQSEMEDLQNQFQKELAEQRAELEKIFQDKNQAERALRNLESHHQAA

IEKLREDLQSEHGRCLEDLEFKFKESEKEKQLELENLQASYEDLKAQSQEEIRRLWSQLDSARTSRQELS

ELHEQLLARASRVEDLEQLKQREKTQHESELEQLRIYFEKKLRDAEKTYQEDLTLLQQRLQGAREDAPLD

SVEVGLSCVGLEEKPEKGRKDHVDELEPERHKESLPRFQAELEESHRHQLEALESPLCIQHEGHVSDRCC

VETSALGHEWHLEPSEGHSQELPWVHLQGVQDGDLEADTEGAARVLGLETEHKVQLSLLQTELKEEIELL

KIENRNLYEKLQHETHLKDDLEKVKHNLIEDHQKELNNAKQKTELMKQEFQRKETDWKVLKEELQREAEE

KLTSMLLELREKAESEKQTIINKFELREAEMRQLQDQQAAQILDLERSLTEQQGRLQQLEQDLTSDDALH

CSQCGREPPTAQDGELAALHVKEDCALQLMLARSRFLEERKEITEKFSAEQDAFLQEAQEQHARELRLLQ

ERHQQQLLSVTAELEARHQAALGELTASLESKQGALLAARVAEMQTKHAADLSALETRHLSSLDSLESCY

LSEFQTIREEHRQALELLRADFEEQLQKKDSLHQTILTQELEKLKRKHDRELQSVRDHLRTKVSTELAGT

VAHELQGVHQGEFGSEKKTALHEKEETLQLQSAQPQPFHQEEKESLSLQLQKKNHQVQQLKDQVLSLSHE

IQECRSELEVLQQRRERENREGANLLSMLKADVDLSHSERGALQDALRRLLGLFGETLRAAVTLRSRIGE

RVGLCLDDAGAGLAPSAAPALEETWSDVALPELDRTLSECAEMSSVAEISSHMRESFLMSPESVRECEQP

IRRVFQSLSLAVDGLMEMALDSSRQLEEARQIHSRFEKEFSFKNEETAQVVRKHQELLECLKEESTAKAE

LALELHKTQGTLEGFKVETADLKEALAGKEDSEHRLVLELESLRRQLQQAAQEQAALREECTRLWSQGEA

TATDAEAREAGTAVTAAHKDSALRKEVEDLTKEQSETRKQAEKDRSALLSQMKILESELEEQLSQHRGCA

KQAEAVTALEQQVASLDKHLRNQRQFMDEQAAEREHEREEFQQEIQRLEGQLRQAAKPQPWGPRDSQQAP

LDGEVELLRQKLREKSDEFNELAIQKESADRQVLMQEEEIKRLEEMNINIRKKVAQLQEEVEKQKNVVKG

LEQDKEVLKKQQMSSLLLASTLQSTLDAGRCPEPPSGSPPEGPEVQLEVTQRALLRHESEVLDLKEQLEK

MKGDLESKNEEILHLNLKLDMQNSQTAVSLRELEEENTSLKVIYTRSSEIEELKATIENLQENQKRLQKE

KAEEIEQLHEVIEKLQHELSLMGPVVHEVSDSQAGSLQSELLCSQAGGPRGQALQGELEAALEAKEALSR

LLADQERRHSQALEALQQRLQGAEEAAELQLAELECNVALREAEVEDMASRIQEFEAALKAKEAMIAERN

LEIDTLNQRKAAHSAELEAVLLALARIRRALEQQPLAAGVEPPELQWLRAQCARLSRQLQVLHQRFLRCQ

VELDRRQARRATAHTRVPGAHPQLRMDGGAKTQVTGDVEASHDAALEPVVPDPQGDLQPVLVTLKDAPLC

KQEGVMSVLTVCQRQLQSELLLVKNEMRLSLEDGGKGKEKVLEDCQLPKVDLVAQVKQLQEKLNRLLYSM

TFQNVDAADTKSPWPMASAHLLESSWSDDSCDGEEPDISPPIDTCDANTAMGGVTDVIKNQAIDACDANT

TTGGVTDVIKNWDSLIPDEMPDSPIQEKSECQDRSLSSPTSVLGGSHHQSHTAEAGPRKSPVGMLDLSSW

SSPEVLRKDWTLEPRPSLPVTPHSGALSLCSADTSLGDRADTSLPQTQGPGLLCSPGVSTAALALQWAES

PPADDHHVQRTAVEKDVEDFITTSFDSQETLSSPPPGLEGKADRSEKSDGSGFGARLSPGSGGPEARTAG

PVTPAPISGRFQPLPEAMKEKEVHPKHVKALLQMVRDESHQILALSEGLAPPSGEPHPPRKEDEIQDSSL

HGGKTQEVPTACPDWRGDLLQVVQEAFEKEQEMQGVELQPRLSGSDLGGHSSLLERLEKIIREQGDLQEK

SLEHLRLPDRSSLLSEIQALRAQLRMTHLQNQEKLQHLRTALTSAEARGSQQEHQLRRQVELLAYKVEQE

KCIAGDLQKTLSEEQEKASGVQKLLAAEQTVVRDLKSDLCESRQKNEQLSRSLCEVQQEALQLRSMLSGK

ENELKAALQELESEQGKGRALQSQLEEEQLRHLQREGQSAKALEELRASLETQRAQSSRLCVALKHEQTA

KDNLQKELRIEHSRCEALLAQERSQLSELQKDLAAEKSRTLELSEALRHERLLTEQLSQRTQEACMHQDT

QAHHALLRKLKEEKSRVVDLQAMLEKVQQQALHSQQQLEAEAQKHCEALKREKEVSATLKSTVEALHTQK

RELRCSLEREREKPAWLQAELEQSHPRLKEQEGRKAARRSVEARQSPAAAEQWRKWQRDKEKLRELELQR

QRDLHKIKQLQQTVRDLESKDEVPGSRLHLGSARRAAGSDADHLWEQQRELEAMRQRLLSAARLLTSFTS

QAVDRTVNDWTSSNEKAVMSLLHTLEELKSDLSRPTSSQKKMAAELQFQFVDILLKDNVSLTKALSTVTQ

EKLELSRAVSKLEKMLKHHLQKGRSPSRSERSAWKPDETAPQSSLRRPDPGRLPPAASEEAHTSNVKMEK

LYLHYLRAESFRKALIYQKKYLLLLIGGFQDSEQETLSMIAHLGVFPSKAERKITSRPFTRFRTAVRVVI

AILRLRFLVKKWQEVDRKGALAQGKAPRTGPRARRPQSPPRTRESPPTRDVPSGHTRDPARGRRLAAAAS

PHSGGRATPSPNSRLERSLTASQDPEHSLTEYIHHLEVIQQRLGGVLPDSTSKKSCHQMIKQ

>MACACA MULATTA

DGSHSEKKTAKRKGSAVDAPVQEESPIAKEDRALRGGGDIC

KSTSCDDTPDGAGGAFAAQLEDRDGEKREDLEQLQQKQVSDHPPEQRGIFTVSDPCQEQRGIFTVSDRHP

EQRGMFTVSDHPPEQRGMFTVSDPHPEQRGMFTVSDHHPEQRGIFTVSDPHPEQRGIFTVSDPHPEQRGI

FTVSDPHPEQRGIFTVSDPHPEQREVITKECKQEFELAIADLQSSREEEAGLQQSQAVHGLELEALRLSL

SNMHTAQLELTQANLQKEKETALTELREMLNSRRAQELALLQSRQQHELELLREQHAREKEEVVLRCGQE

AAELKEKLQSEMEKNAQIEKTLKEDWESEKDLCLENLRKELSAKHQSEMEDLQNQFQKELAEQRAELEKI

FQDKNQAESALRNLESQHQAAIEKLREDLQSEHCRCLEDLEFKFKESEKEKQLELENLQASYEDLKAQSQ

EEIRRLWSQLDSARTSRQELSELHEQLLARASHVEDLEQLKQREKTQHESELEQLRIYFEKKLRDAEKTY

QEDLTLLQQRLQGASEDALLDPVEVGLSCVGLEEKPEKERKDHVDELNPERHKESLPCFQAELEESHRHL

LEAPESPLCIQREGHVSDRRCAETSALGQGWRLEPSEGRSQGLPWVHLQGVQDRALEADTEVAARVLGLE

TEHKVKLSLLQTELKEEIELLKIENRNLYEKLQHETRLKENLEKVKHNLVEDHQEELNNAKQKTELMKQE

FQKKEMDWKVMKEELQREAEEKLTLMLLELREKAESEKQTIVNKFELREAEMRQLQDQQAAQILDLERSL

TEQQGRLQQLEQELTSDDALRCSQCGREPPTAQDGELAALHVKEDCALQLILARSRFLEERKEITEKFSA

EQDAFLREAQEQHTRELQLLQERHQQRLLSVTAELEARHQAALRELTASLESKQGALLAARVAELQTEHA

ADLSALETRHLSSLDSLESCYLSEIQTIREEHRRALELLRADFEEQLRKQDSLHQTILTQELEKLKQKHD

EELQSVRDGLQTAASTELAGTVAHELQGAHQGEFGSEKKAALHEKEKTLWLQSAQAQPFHQEEKESLSLQ

LQKKNHQVQQLKDQVLSLSHEIEECRSELEALQQRRERENREGANLLSMLKADVDLSHSERGALQDTLRR

LLGLFGETLRAAITLRSRIGERVGLCLDDAGAGLALSAAPAQEETWSDVALPELDRTLSECAEMSSVAEI

SSHMRESFLMSPESVRECEQPIRRVFQSLSLAVDGLLEMALDSSRQLEEARQIHSRFEKEFSFKNEETAQ

VVRKHQELLECLKEESAAKAELALELHKTQGTLEGFKVETADLKETLAGKEDSEHRLVLELESLRRQLQQ

AAQEQAALREECTRLWSRGEATATDAEAREAGTAVTAAHKDSALRKEVEDLTREQSETRKQAEKDRSALL

SQMKILESELEEQLSQHRGCAKQAEAVTALEQQVASLDKHLRNQRQFMDEQAAEREHEREEFQQEIQRLE

GQLRQVAKPQPWGPHDSQQTPLDGEVELLQQKLREKLDEFNELALQKESADRQVLRQEEEIKRLEETNIN

IRKKMAELQEEVEKQKNIVKELAQDKELLKKQQMSSLLLASTLQSTLDAGSCPEPPPGSPPEGPEVQLEV

TQSALLQRESEVLDLKEQLEKMKGVLESKNEEILHLNLKLDLKNSQTAVRLGELQEESVSSKVVYTRSSE

IEELKATIENLRENQKRLQKEKAEEIEQLHEVIEKLQHELSLMGPVVHEVSDSQAGSLQSELLCSQAGGP

RGQALQGELEAALEAKEALSRLLADQERGHSQALEALQQRLQGAEEAAERQLAELERSAALREAEVEGMA

SRIQEFEAALKAKEAMIAERNLEIDALNQRKVAHSAELEAVLLALAHIRCVLEQQPLAAGAEPPELQRLR

AQCARLSRQLHVLHQRFLRCQVELDRRQACRTTAHTRVPGAHPQPRVDGGAKAQVPGDMEASHDGALEPV

VPDPQGDLQPVLMTLEDAPLCKQEGVMSVLTVCQRQLQSELLLVKNEMRLSLEDVGKGKEKVLEDCQLQK

VDLIAQVKQLQEKLNRLLYSMTLQNVDAADAKSPWPLASAHLLESSWSDGSCDGEEPDTSAPTDACDASA

ATGGVTDVIKNRDSLIPDEMPDSPIQEKSECQDRSLSSPTSMLGGSCHQSHAAEAGPRKSPVGMLDLSSW

SSPEVLRKDWSLEPRPSLPVTPHSGALSLCSADTSLRDRADTSLPQPQGPGLLCSPGVSAAALALQWAES

PPADDHRVQRTAVEKDVEDFITTSLDSQETLSSPPPGLEGKTDGSEKSDGSGFGARLSLGSGGPEAPTAG

PVTPAAIPGRFQPLPAAMKEKEVHPQHVKALLQMVCDESHQILALSEGLAPLTGEPHPPRKGDKDVPTVC

PDWRGELLQVVQEAFEKEPEIQGVELQPRLSGSDLGGHSFPLERLEKVISEQGDLQEKSLEQLCLPDRSS

LLSEIQALRAQLRMVHLQNQEKLQHLRTALTSAEARGSQQEHQLRKQVELLVYKVEQEKCIAGDLQKTLS

EEQEKANGVQKLLAAEQSVVRDLKSELCESRQESERLSRSLCEVQQEVLQLRSMLSSKENELKAALQELE

SEQGKGRALQSQLEEEQLRHLQREGQSAKALEELRASLEGQRAQSSRLCVALKHEQTAKDNLQKELRIEH

SRCEALLAQERSQLSELQKDLAAEKSRTLELSEALRHERLLTEQLSQRTQEACVHQDTQAHHALLRKLKD

EKSRVADLQAMLEKVQQQALRSQQQLEAEAQEHCEALRREKEVSATLKSTVEALHTQKRELRCSLERERE

KPAWLQAELEQSHPRLKEQEGRKASRRSTEAGQSPAAAERWRKWQRDKEKLRELELQRQRDLHKIKQLQQ

TVRDLESKDEVPGSRLHLGSARRAAGLDADHLREQQRELEAMRQRLLCAAGLLTSFASQAMDRTVNDWTS

SNEKAVVSLLHTLEELKSDLSRPTSSQKKMAAELQLQFVDVLLKDNVSLTKALSTVTREKLELSRAVSKL

EKMLKHHLQKGCSPSKSERAAWKPDETAPQSSLRRPDPGRLPPAASEEAHPSNVKMEKLYLHYLRAESFR

KALIYQKKYLLLLIGGFQDSEQETLSMIAHLGVFPSKAERKTTSRPFTRFRTAVRVVVAVLRLRFLVKKW

QEVDRKGALAQGKVPRPGPRVRRPQSPPRTRESPPTRDVPSGHTRDPARGHGLAAAASPHTGGRATPSPN

SRLERSLTASQDPEHSLTEYIHHLEVIQQRLGGALPDSPSKKSCHQKIKQ

>BOS TAURUS

KGDCTHSKKTPAKRKGTTVNAPVTEESPVAAPGNTGPLGGLD

IGKNPTCSDTPDGAEAAQLQEPSRERTPPKLDGHCAEQPGVMTKELELEVLRLSLSDMHAARLERTQAHL

QREKEAALAELHAELTGRHAQERALLQSRARRELELVREEAAELKEKLQSEIEKNAQMMEILKQDWESER

DSCLENLRQELLEKHRSELENLQNQFKKELAQQKAEFEKVFQAKNQAECSLQTQEAQHEASLSKLRADLQ

LEHCQYLEELERKFRKKEKENQLELERLRASYEELQAQSREEIQCLGAQLESARSSRQELSELREQLLAR

ASHVEELELLKRDLEQQQQQERSRHESELEQLRLYFEEKLREAEQSYQEDLTLLQRRLQEGRDDSLLEAM

GTSACLEETSEEERDHLNGLSLQREQLEESLGLRTRLEENHQCQLAAGTSSQDAQQEALEGELPTGPSEE

PGQELAREPLLCAQSVALELETEVQAGDMGLETAHRAQLVLLPVELKKEIDLLKVENRNLQEKLQREICL

KEDLEKVKQNLVEAHQEELKSAEERIEQIKQELREREAEWKVTSEDLRRGAEEKLTKMLLDLREQVESEK

RSMMNRFELRESEMRQLQDQQAAQILDLEGSLVEQQGRLRQLELGLTGDESLRCSHCGRELGCSPAPGAQ

DQELAILHLKEDCALQLKVAQSRFLEERRAITEKLAAEQDAFLREAQEQHAHELRLLQEGHQRHILSLTA

ELETRHQAEVDALRSSLERERLALAEAREAELQVKHAADINVLEARHASHLDALRLQYLSELQAVQGRHR

QALELLRLDLEERLQKEDMAHHAVLTQVLELLELRCAEEPQPAEDSLRGDGSVEPQEGVRAVRLSMAHKE

PEAQCSAPQAGQEEELELLERKPEASLEQQVALLQGEFGSERKAALHGKEEIHKLDHEKAHSLYEKEKES

LSLQLQEKNNQILQLKDQILSLNREVEERHSELEKLQERRERENQEGTALIAMLKSDVDLSHGERRALQD

TLRRLLGLFGETLQTAIALKSRIGERVGLCLEDRSPPTTQPGGQSPPTDPALDDMWAGLDVASLEPDGTV

AECAETSSVAEISSHVCESFFLSPERTLDCEQPVRRVFQSLGLAVDGLLEMVLDSARQLEEARQIHSRFE

KEFSCKNEETAQVVRKHQELLEQLEAESKAKAQLALELHKAEGIIEGFKEEKAGLQEALGQKEASERGLV

GELEGLRQQLQRAARRQAELREENAALCSQTEALAADAREREAGIPVSVAHEDSALRREVESLTQEQSEA

RRQADKDRASLLSQMRFLEAELEEQLSHQQACARQAEELAALRQQIDSLDQHLRRQRQFMDEQAVERERE

REDFQQEIQRLEEQLRQAARPRSPGLRDSHRQRAQLDKEVELLEEKLREKSDGLNELILKKELVDRQVLI

QGEEIRRLEETNASTTRKLLQLQEELETQRRAVRALQQDKEALQEQQMSNLLQVSALQSRLDEGKCPVPP

EGGRPDRAEAQLEAMQGALRQREAEVSDIKEQLENLKDDLVNKNEEILHLNWKLGLQDNSTAASIKELQE

ENAHLKTLLQNKESEMMHVRESAEAQLARTGSGALSEVVCNRSSEVEELKSIIETLRENQARLQKDNAEE

IEQLHEVIEKLQRELPLGGPEAPKAGEGGAMSLQSELLGLPAEGAEAPAVLAGELHAALEAKEALSQRLA

EQERQHSQALEALQQRLQAAEEAATRQLAELQPEGVASQIREFEAALHAREAKIAERDLEIQALRREKST

HSAELEAILAAVARFRHALEQQLLAASGEPPELQRLREQCVCLSRQLQALNQRFLKCQKELDERQACGEG

GSQGWVPRGKETSCSEELQQDVDGRQLAQALDSRGSDPQSLVKDDLQPAKALPTLTCARPSQQDSVISVL

TVCQQQLESELLLVKKEMRLHAEDGGRASGAVKDKGKLLEDCRLQKVDLIAQVKQLQEKLNHLVCSMSFQ

NIEMEDFKYQQALASPCALEDGLSDGSDNSEETDKSPPIDTFNIIWDLAEVTGNSDSLIRNEPDAPTGEQ

VTLQDRSLCLQSHLHGCSQDLTHSQGSKPLKNVLRTMGLSPWSSPEVVREDSTLEPLPNLPLTPCSDALS

LHSLDTSLWDGTSTHVLQADQSGRLCYPGESAAGTAPKWVGSPLAADGVPSANHHAQRVPVEKDVEDFII

TSLDSQEKSRSPPLGLEGKSDGKSQLRSCGRTARLHPVPSEQPSVLGGGVEFAEPLSQGSGRLGAPTASP

AAPPVTPRDCRRPLDTMKEKEVHPKQVKALLQMVCDESHHILALSEYREPPHILSRSEPSARLECLPLGG

QGLLEAVPAPREHPTPGSQKGEKASSNVGLDWKGDFLQTVQEAFEKEWEMLRVELWPQLCGLGPGGCGSL

LERLEKVVHEQGDQQERSLEHLCLSDRSSLLSEIQALRAQLRMTHLQNQEKLQQLCTALTSAEARGSRQE

HRLRRQVELLAYKVEQEKCIASDLQKTLNEEQEKANNVRKLLVAEQNAVRDLKSELCECRQDNERLLKSL

NDVQKEVLQLRSMLDSKESDLQAALQHLEGERMKEQALQSQLEEERLQHMQREGQSAKTLEELRASLEQQ

CAQSNQLCVVLKHEQTAKDNLRQELQIEASRCEALLAQERGHMSELQRSLEAERDRARELSEALQHERIL

TERLSRRPQERPAHQALLQKLRAQEEHVRELEVALEAAQRRALEAEAHVHREEVEREQEVSAAPKTPPET

LPGTRERPARGRGRVEQDACEDVRTGAELRPSRADTQPWSRWQRDKETLRELELQRQRDEHKIQQLQRTV

QELQARVALPNTEPEHLREQQRGLEKVRQQLLCAAGLLTSFINQTVDRTISDWTSSNEKAVASLLHTLET

LKSELGTSSSCRKKTAAELQIQLVDVLLKDNESLSRALSTVTREKAELCKALSRLERAHRVPRTLQEGCA

RKRSDRSAWKQDKMVSPGSARHPDPALDAGACSAKMEKLYLRYLRAESFRKALIYQKKYLLLLIGGFQDS

EQETLSMIAHLGVFPSKADKKAAGPRPLTRFRTAVRVVIAVLRLRFLVKKWQQVDGRGALAPGQALRPGE

GFLVPRRQQSPPVTRESPPTRDVSSSQARDPVPKASPRRREKSSPPSNPRSERSLTASQDPEHSLTEYIH

HLEMIQQRLGGVLPDSTSKKSCCQKMKQ

>CANIS LUPUS FAMILIARIS

MDEDEQEQRRRKVEAGRAKLAHFRQRKTKGDCAYPKKKTAKRKGPAVDAPVQEESPVATEDGGLLGRGGD

VCRDTSCSDTPDGAGAAQDREQDCKLEIAQPMGRQEDEAGAQQQVAVELRQPAGMQQVQLQTQPVPSLEL

EALRLSLSNMHTAQLELTQANLQKEKETALTELRAMLNGRHAQELALLRSRQQQELELAREQHAREQEEM

RLRCSQETAKLKEKLQSEMERNARAVESLKRDWESERGLCLENLRRELSAKHRSELENLQDQFKRELSEQ

KAELEKIFQAKNQAECALRTLEAQHEEALRQLREDLQSERCQYVQDLELRLRDQEVEKQLELESLRASYA

ELKAQSQEEIRRLWSQLESPRPDRQDASARTPHGEELEQRERECGVQQQRRGRRAEQESELGQSPEGAAE

RGREDPVLLEQRLQDATEEPSLESGGIGPSSMREGERREQRVGPSLQPELAENHRCHLTAWTSSLEAQQQ

AGLVETQESEGEQREGLSEGPGQELTQVPVPCAQGTALKVDTEVVARGLGLEAEHRLKLLLLQTELKEEM

DVLKLENRNLHEKLQHEVCLKEDLERVKHSLVEDHREELKKANERLQLVKQELKDKEAEWKVASEDLKRK

AEEELTLMLHDLKEQAEAEKQSVINKFELREIKMRQLQDQQAAQILDLEGSLVEQQGRLRQLEHGLEAEE

CPRCGHCGQEPAGRVAPADQDWELATRRLREDCALQLMQAQSRFLKERKELTEKFTAEQDALLQEAQEKH

ACDMRLLQERHQQHVLSLTAQLEARRRAEVEELKASVESERWALAEARVAELQTEHAAAISALEARHSAL

LGSLESRHLSEVQAMREEHRQALEQLRVDLEKQLQEKEASHPTPLTQALEKHPLSHGQELPPVEGGLRTQ

TSSGQLEGVKAPVPPEVQGAQQEGAPVPAEVEAQRPALLAELRQETGKQQLHCQEEPEVLECPSEPFLEQ

QILQPKGEFESEKKVALHEKEIHGLECAQAQSLYQKEKESLFLQLQEKNNQILQLKEQILSLSHEVEERR

RDLEQLQQRRERENQEGTNLISMLKSDADLSDRERRALRDALRRLLSLFGEMLKVAIALKSRISERVGLC

LEDEDPPGAWPSGQALSAAPVLEETWPGSEAALLELDRSLPECVEMPSEAEISSHICESFFMSPESSLEY

EQPIRRIYQSLGLAVEGLLELALDSTRQLEEARQIHSRFEKEFSCKNEETAQVVRKQQQLLERLEEENAA

RTRLMLELHQAQGIIEGFKEEKASLQEALCRQETAEHGLAEELEGLRQQLQQAAQQQAELKEENSALWSQ

KEASAAEAEAREAGTPAPIAHEDSALRREVECLTQEQSEARKQSEKDRAALLSQMKVFEAELEEQLSRHE

ACAKQAEELSALRQQMAALDKHLRSQRQFMDEQAVEREHEREEFQREIQRLEEQLRQAARPRPHGPRVSD

EEQLDEEVELLQEKLREKSDGFNELVIKKELADRQVMIQEEEIRRLEETNASCRREAAQLREELERQRDA

MKALQQDKEALQEKQMSNLLLVSTLQSKLDEGRCPVPPADSCPEGPEVQLEAVQRALQQRESEVLDLKEQ

LGKMKDDLVSKSDEVLHLNLELDAQSSRAAVSVRELQEENASLKAFLQNKEKEIMCVSEQLEAQLAGMGS

GALREVTYDRSSEIEELRSIIENLRENQQRLQKEKAEEMEQLHEVIERLQEELSLGAPAVPAAVDCELPA

VPAPAVGPEALAAAGAASRLFAEQEHRHGQALEALQQRLQAAEEAAAGQLAELERSAALREAEVQAMASQ

IQAFEAALRAKEARLAERDLEIDAMKRQKLAHSAELETILAAFSRFRRTLERQPLAAEDEPPELQRLRVQ

CVRLSRQLQVLNQRFLRCQKEADKQQARGARLRPRGARGLQGPGPRAEEASRDEASQQDVDSRQVASATQ

GQVRDPQNAVNDDLQPAELPVTSGPPGLLKQDSVMSVLAVCQRQLESELLLLKNEMHLRTEDRGKAPARM

KNKENLLEDRQLQKVGLISQVKELQEKLNHLVRSVNFQNIETEDFKSQQPVACSRALENSLSDSSSNGEE

TDRSSLVDAFHIDKTTCDLNDLSGNQDPLMRNEMLDVPVEDKVELQEGPRCLQANFHGGSPDLTPTEGPG

PLKNALRAMDLSSWSSPEVVRKDLTLEPVPSLPLTPCSDALSLDASLQDRTSASLQADQLELLWYLGQSP

AEKASRSAESPLAADRVPSADHHVLQMAVEKDVEDFIVTSLDAQEKPRSSPLGFEGKSNGSENSDGSACG

EILNQDLGRLGAPAARPPGPPAASGRFRRPLEAMKEKEVHPKQVKALLQMVCDESHHILALSECRGLPSA

LSRGEPRAPLEHFPGEGPGLLEAVPAPRRHPHLAPQKGEQEPPDPGLDWRGEFLQAVQEAFGKEREMLRA

ELQPRPCGSAPGEHSSLLERLEKVVQEQEKSLEHLRASDRSSLLSEIQALRAQLRMTHLQNQEKLQQLCA

ALTSAEARGSRQEHQLRRQVELLAYKVEQEKRIASDVQKTLTEEQEKASSVRKLLVVEQTVVRDLRSELR

ECKQDNERLLASLGEAQKEVLQLRSVLDSKESNLRAALQELESERGKERALQSRLEEAQLQHLQREGQSS

KTLEELRASLEKQFAQSSQLCVALKHEQTAKDNLQKELQIEASRCEALLAQERGQLSELQRSLEAEKGRS

LELAAALRHERLLTEQLSRGPRESPAHHALLRKLKEEKSRAAELQARLEQVQRQQEADVQKRRAELEREK

EVRAAQAREPGRCLRREGHASQQQAGLEPLQAGLAAQEGRKDARRADVGPGRADAEMRPTGAKEKLRELE

LQRQRDEHKIRQLQRTVQALEAKEEAAQRPEAERLQEERLGLERVRQQLLCAAGLLTSFISRTVDRTISD

WTSSNEKAVTSLLHTLEELKSELSTPSSSQKKMTAELQTQLVDVLLKDNDSLTKALRTVTQEKAELCRAV

SQLEKSLKHHVLRRGVCSKPDRCAGKQDGTALPSSARQPDPGLPPAAAREDANTCGVQMEKLYLHYLRAE

SFRKALIYQKKYLLLLIGGFQDSEQETLSMIAHLGVFPSKADKKTTTSRPFTRFRTAVRVVIAILRLRFL

VKKWQEVDRRGAATHGRAPRPGLPAPRQQPPPAASRSPPTRDTSCSRSGDRAPKASPGRRHRGNHASVYS

GAGTREAVGAVSFPPRSPRICNKAHLSLCLRGVGTCVWNPPCQRVPLSAPASTPSPDSRSQGSPAASQDP

ERSLTEYIHHLEVIQQRLGGAAADSGSKKSCCQRMKQ

>RATTUS NORVEGICUS

MEDEQEQRRRKVEAGRAKLANFRQRKTKGDGPNSKKKTAKRKGSAVHASVQEEGSVAPPNSELPQGAAVS

ERESCGNTLEGTGGAFAAQEQQDCELGVTDLQGQQQPQQQPQPPQTAHSLELEALRLSLNNMHTAQLELT

QANLQKEKETALTELREMLNGRRAQELALLQSRQQCELELMREQHAREKEEMALRNEQETAELKEKFRSE

MEKTVQMMETLKQDWESERELCLETLRKELSAKHQSEMEGLQNQFQKELSEQKAELEKIFQAKHEAEVSL

KNLEAQHQAAIRKLQEDLRSEHCQYLQDLELRFREKEKAKELELETLQASYEDLKAQSQEEIRHLWSQLE

SMKTNREEQNGSWEPLLAQASHLEELQHLRSDFAQQQQQERAQHESELEHLRVYFEKKLNDAEKTYQEDL

TVFQQRLQEAREESLESAEISSSCMFPEETSGRERKEPPDPLDLQLEQPKAQGSLIEDYQEKLSNAEEKI

ELMKQEFQKKEAEWELSREDLKRDAEEKLASMFLELREKAESEKLSIINRFELRESSMRHLQDQQAAQIS

DLERSLREQQGHLRQLEQELTRDEVLLCSQCGKEPSVAQDEKSAILLREKEDCALQLLTAQNRFLEERKE

IMEKFAKEQDAFLLDTQEKHSHELQLLQQGHQQQLLALRMELETKHHSELTEQLASLESKQQALLETHVA

KMQVKHDAEISALEKRHLSNLDELESCYVADIQTIRDEHKKALELLRAELEEQLQKKDSCHREILTQELE

KLKLKHAEELQSVRNSLRVKMSAQHTESGKGPAADLQGAHQQKAPAMALHNEGHQLEEDGDAALGGADTE

DLQHHAEPREREGPHTVEMQTSQTELAKPQELQASQDQGAQVRDQVFLLSRELEECRGELEQLQQRRERE

NQEGATLICMLRADVDLAQSEGKALRDALRRLLDLFGETLKAAVTLKSRISERAGLLLDHEDAVDTSGAQ

LAAAALGEMWPDEGLLDIDRTLPEGAETSSVCEISSHVCESFFMSPENTQECEQPIRKVYQSLSTAVEGL

LDMALDSSKQLEDARQLHRCVEKEFRHRDEEMAQAVQKQQELLERLREESAAKDRLALELHTAEGLLEGF

KVEKADLQEALGKKEESEQQLIVELEDLRKQLQQASRELLTLKEENSVLWNQKETFTNEAKEREAGTPVS

AGREDAALQKEVESLTRDQWESRKQSEKDRATLLSQMRVLESELEDQLVQHRGCAQLAEEVTTLKQQLAT

LDKHLRSQRQFMDEQAAEREHEREEFQQEIKWLEGQLRQATRPRPPGPRDSQCAKLDEEVELLQEKLREK

LDGFNELVIKKDLADQQLLIQEEEIKHLEETNENIQRQMAQLQEELEKQRRSTEELEVVNSRNSEIEELK

AIIEHLQENQEQLQKAKAEEIEQLHEVIEKLQSELSLMGPTVHEMSDLPPGSLHTELSCLQAEGMGGQAL

HNELQAAQAAKGAFGQLLADHGHSQALEALQERLQDAEAAAARHLTELEHCVALRQAEVEAMASRIQEFE

ATLKAKEAIIVQRDLEIDAVNKWKVSHSVELEALLLALAHFQHAVEQQTSATPDEPPKLQQLLVQCARLS

HQLHVLYRRFLRCQVELGQHQPRVASVGCADPPAEAQGKRDGELEQDGVSSGLALAPHSLVAQAKEEQPE

DCHLGNTSLRAQLRQLEEKLDPQAHSVAFRDTSMETCKLQQPNPLENGSSNHCHNGEESRPSPPNDVPAK

TTWDVIGVIKNQDLLAQIEMPDSPTPEKLASQGGAFSSQASVHGASLLPEEAEPQKDPVRALDLSSWSSP

EVLRKEPSLEPQHSLPLTPGVGTVSLHSVDISSPDWTDPLLQADVSGLLCYPGKSATGQAPLWVVAPSAE

KHHVERTATEKDVEDFIVTSFDSQESLTSPSHELARRSDGSGKSDGPDIAAMLTLGSEGSETPTTDLVAA

TTGPFSRRFVQSPGAMREKEVHAKQMKALLQMVFDESHQILALSESQDSSSALSKGEPRDPLDGVPRDRQ

ALSEGTSDRGEKEALGTHLTWSEELLQAIQEVFAREQENAELQPRPYSSDLGDYNSLVKKLEKVIQEQGD

LQKVREHACLPDRSSLLAEIQALRAQLRMTHLQNQEKLQQLCAALTSTEARGSQREHQLRRQVELLAYKV

EQEKCIANDLQKTLSKEQEAASDVRKRLMVEQGAVQDLKSELHACKQENTNLLESLDKVQQEVLRLRYGH

VTWSYSGTSSASAGRAVLDGKENELKVVLEELECERGKGQVLQAQQEEQQLRHLQREGQSSRALEELKIS

LEKQLAQNNQLCVALKHERAAKDNLQKELQIEASRCEALLAQEKGQLSELRKSLEAERSRSLELSEALQH

ERLLTEQLSRNAQEACARQDTQAQHALLRKLKAEKARALELEAMLEKVQKQAAHTQQQLEAQAQERCLEL

RREKEVSGNLRSAVDALQTHKQELGCCLEREREKAAWLQAELEQLSARVKQQEARQDEKRMDRRSSREDL

DKRKWQRVKEKLRELELQRQRDEHKIEQLQRLVRELRWKEEAPGGNGPCRGSPCLGSLERDQFQEQQQEL

EKIRQQLLCAAGFLTSFTNHTVDRTIKDWTSSNEKAVSSLMRTLEELKSELSMPTSFQKKMAAELQVQLM

NELLSDNDALTKAVGMATREKAELCKTVSRLEKTLKHHTQKGCILNRQSKTPLKQDGMDLQSSLPHSDPE

WHPQTTGGDTNTCNIKMEKLYLHYLRAESFRKALIYQKKYLLLLIGGFQDSEQETLSMIAHLGVFPSKAD

KKVTASRPFTRFRTAVRVVIAVLRLRFLVKKWQEVDRKGALIHHSRSTRHGHRTSQRQNSPSGTRASLPT

RDVSSGHTKDSRHSPKCSATSLGKGRSTSSTSSRLERSLTASQDPEQSLTEYIHLLEMTQQRLAGLPPDA

PQKACPQKIKQ

>MUS MUSCULUS

KGDCPNSKKKTAKRKGSAVHASVQEEGSVATPNSELPQGGAVF

ESPSCSNTLEGTRGASAAQEQEDCELDVTDLQGQQQTQPPPPQTAHSLELEALRLSLNNMHTAQLELTQA

NLQKEKETALTELREMLNGRRAQELALLQSRQQCELELLREQHAREKEEMALRSGQEAAELKEKLRSEME

KNAQTIETLKQDWESERELCLENLRQELSLKHQSEMEGLQSQFQKELSEQKVELEKIFQAKHEAEVSLKN

LEAQHQAAIKKLQEDLQSEHCQYLQDLEQKFREKEKAKELELETLQASYEDLKAQSQEEIRLLWSQLESM

KTNREELNGSWDPVLAQASHLEELEHLRSGFAQQQQQERAQHESELEHLRVYFEKKLKDAEKTYQEDLTV

FQQRLQEAREDSLESTEISSSCVLPEETSGREGKEPPDPLDLQLGQPKVQESLVEDCQVKLSKAEEKIQQ

MKEEFQKKEAEWELSREELKREAEERLASMFLELREKAESEKLSIISRFEHRESSMRHLQDQQAAQILDL

ERSLMEQQGHLRQLEQELTRDDLLPCSQCGQEPAMAQEEKNGALLREKEDCALQLLMAQNRFLEERKEIM

EKFAKEQDAFLRDAQEKHNHELQLLQQGHQQQLLALRMELETKHRSELTEQLASSESRRQALLETHVAEL

QVKHNAEISALEKRHLSNLDELESCYVADVQTIRDEHQQALELLRAELEEQLQKKESCHREMLTQELENL

KRQHAEELQSVRDSLRMEMSAQHIENGKGPAADLQGAHQQDPAMALHNEGHLLVEDGDAVLRSVDAEGLL

HQAGPQELGDAHTVEMQKSQAELAKPQELQASQDQVAQVRDKVFLLNRELEECRAELEQLQQRRERENQE

GTTLICMLRADLELAQGEGKALRDALRRLLDLFGDTLKAAVTLKSRISERAGLLLDHEDAADTSDARLAA

AALGDMWSDEGLLEIDRTLPEGAETSSVCEISSHVCESFFISPENTLDCEQPIRRVYQSLSTAVEGLLEM

ALDSSKQLEEARQLHRCVEREFRHRNEEMAQAMQKQQELLERLREESAAKDRLALELHTAKGLLEGFKVE

KVDLQEALGKKEESEQQLILELEDLRKQLQQAARELLTLKEEKSVLWNQKETLTNEAKEREAALQEEVES

LTRVQWESRKQSEKDRATLLSQMRVLESELEDQLVQHRGCAQLAEEVATLKQQLAALDKHLRSQRQFMDD

QAAEREHEREEFQQEIQRLEGQLRQAARPRPPGPRDSQCVQLDEEVELLQEKLREKLDGFNELVIKKDFA

DQQLLIQEEEIKRLEETNASIQRQMVQLQEELEKQKKSMEELKEKEILKQENMGDLLLTTVSRSGLDEAG

CPMLPQGSSSRGPEAQPDVTERALLQHENEVVHRRNSEIDELKSLIENLQENQRQLQKDKAEEIEQLHEV

IEKLQSELSLMGPKVHEVSDPQAGSLHSELACLRGEGLGGQALRSELQAAQAAKEVFGQLLADQAHGHSQ

ALEALQQRLQDAEEVAARHLAELEHCVALREAEVEAMASQIQEFAATLKAKEAIIEQRDLEIDAVNKWKV

SHSLELEAILLALAHFRHALEQQTCATPDEPPELRQLRVQCARLSHQLQVLYRPFLKCRMQLDQHQPHVA

SIGCANPCADDELEQEGVSNRLALAPHSLAAQAKEELEDCPLGKANLMAQVRQLQEELDHRVHSVASRDT

NSETCKLQQPNLSENGPRNHCCNGEESKPSPPDDVLNIAKTTWDVIDIIKNQDLLVQVEMPDFPTQEKLT

SQGGPFSSQASGHSGSLLPEEAAEPQQDPVRALDLSSWSSPEVVRKDPSLEPQHSLPLTPGVGTVSLHSV

DISPDWTDPLLQADVSGLLCYPGKSASGQAPLWAVAPSAGKHHAERTATEKDVEDFIVTSFDSQELLTSP

SHELARRSDGSRKSDGPDIAMMLTLGSEGSETPTTDLVAAAAAAVPFSRRFVQSPGAMKEKEIHAKQMKA

LLQMVFDESHQILALSESQDPSSALNKGEPRDPLDGFPRDSQALSEVTTDKGEKESLETHLTWSEELLRA

IQEVFAREQEKAELQPRPYGSNLGDYNSLVQRLEKVIQEQGDPQKVQDHLCLSDRSSLLAEIQALRAQLR

MTHLQNQEKLQQLCAALTSTEARGSQREHQLRRQVELLAYKVEQEKCIANELQKTLSKEQETASDVRKRL

VVEQNAVQDLKSELHACKQENTSLLESLDKVQQEVLRLRYGHVTWSYCGTSSASAGRAVLDGKEKELKVV

LEELESERGKGQALQAQQEEQQLRYLQREGQSSRALEELKLSLEKQLAQNNQLCVALKHERAAKDNLQKE

LQIEASRCEALLAQEKGQLSELQKSLEAERSRSLELSEALQHERLLTEQLSRNSQEACARQETQVQHALL

RKLKAEKTRALELEAMLEKVQKQAAHTQQQLEAQAQERCVELRREKERELEIQRQRDEHKIEQLQRLVRE

LRWKEEVSGGNGPCRGSPGRGSLERDQFQEQQQELEKIRQQLLCAAGLLTSFTNHTVDRTIKDWTSSNEK

AVSSLMRTLEELKSELSMPTSFQKKMTAELQVQLMNELLSDNDALTKAVGMATREKAELCRTVSRLEKTL

KHHTQKGCVLNRQSKSSLKQDGTDLQSSLRHSDPEWHSQTTSGDTNTCNIKMEKLYLHYLRAESFRKALI

YQKKYLLLLIGGFQDSEQETLSMIAHLGVFPSKADKKITMSRPFTKFRTAVRVVIAVLRLRFLVKKWQEV

DRKGALVHPKSTRHGHRTSQRQRSPSGPRASLPTRDTSSGPTKASRHSPRSAAAGSPGKERSTSTPSSRL

ERSLTASQDPEHSLTEYIHHLEMIQQRLGGLPPDSTQKSCHQKIKQ
